# Data set on coping strategies in the digital age: The role of psychological well-being and social capital among university students in Java Timor, Surabaya, Indonesia

**DOI:** 10.1016/j.dib.2020.105583

**Published:** 2020-04-23

**Authors:** Mr. Ansar Abbas, Prof. Anis Eliyana, Dr. Dian Ekowati, Mr. Muhammad Saud, Mr. Ali Raza, Ms. Ratna Wardani

**Affiliations:** a Department of Management Sciences, Faculty of Economics and Business, University of Airlangga, Surabaya, Indonesia; b Department of Sociology, Faculty of Social and Political Science, University of Airlangga, Surabaya, Indonesia; c Department of Economics, Faculty of Economics and Business, University of Airlangga, Surabaya, Indonesia

**Keywords:** Technostress, Techno-overload, Techno-Anxiety, Techno-Complexity, Psychological well-being, Social capital, Coping strategies

## Abstract

The data article investigates the role of coping strategies, psychological and social well-being in the time of stress due to the effects of technology. Increased technology in the life of students introduces complexities, uncertainty, and overload in higher education institutes. This data provides an ideal research scope for examining the effects of coping strategies on social and psychological well-being. The present dataset includes three hundred and one (301) survey questionnaires from university students in Surabaya city, Java Timor province, by using simple random sampling techniques. This article includes information on reliability and factor loadings, as well as results of regression analyses.

Specifications table

**Table:** 

Subject	Human Resource Management

Specific subject area	Management, Human Resource Management
Type of data	Tables and Figures
How data were acquired	Survey Questionnaire (questionnaire included in Mendeley repository)
Data format	Raw, analyzed
Parameters for data collection	The respondents of this article were exclusively university students and are currently enrolled in government universities.
Description of data collection	The data collected in the spring semester of 2019 from Surabaya, Indonesia. An online survey questionnaire was shared with 350 students, generating 301 responses.
Online survey questionnaire	Data source location Airlangga University, Surabaya, Java Timor, Indonesia, -7.250445, 112.768845, 7° 15’ 1.6020” S, 112° 46’7.8420” E, Feb-July 2019
Data accessibility	Repository name: Mendeley data, Data identification number: DOI: 10.17632/jz42th6t4t.5

## Value of the data

• The data can be used to explain how students use coping strategies (e.g. avoidance, seeking support, problem solving, and religious coping) to reduce the stress due to technology overload, complexity, and uncertainty.
• The data is important for policy implementation (e.g., adopting new technology, replacing or including similar technology) in higher education in the digital age.
• The data is also valuable for designing student's psychological and social activities (e.g., constructing students learning through psychological and social engagement, planning and coordinating students’ events) on campus.

## 1 Data

The data can provide insight into the relations between social and psychological well-being of individuals, and coping strategies against technostress (TS) [1]. Structural equation modeling and factor analysis are used to validate the construct, and the relations between coping strategies, well-being, and technology-related stress are analyzed by using regression analyses. Table 1 through 6 present demographic statistics, correlation coefficients, factor loadings, construct validity construct, discriminant validity, and Hetero Trait and Mono Trait (HTMT) analyses, respectively.

**Table 1:** Demographics Table

N=301		Frequency	Percent	Total %
Gender	Male	84	27.9	29.7
	Female	217	72.1	100
Nationality	Indonesian	214	71.1	71.1
	Foreigner	87	28.9	100
Religion	Muslim	157	52.2	52.2
	Hindu	13	4.3	56.5
	Christian	110	36.5	93
	Buddhist	21	7	100
Age	<25	169	56.1	56.1
	25-35	120	39.9	96
	35>	12	4	100
Education	S1 Bachelors	173	57.5	57.5
	S2 Masters	114	37.9	95.3
	S3 PhD	14	4.7	100
Use of internet	Personal Use	36	12	12
	Studies	35	11.6	23.6
	Socializing	80	26.6	50.2
	All the above	150	49.8	100

Note: The six (6) demographic variables were coded in data as Gender (1-Female, 2-Male) Nationality (1-Inodnesian, 2-Foreigner) Religion (1-Muslim, 2-Christian, 3-Hindu, 4-Buddist) Age (1-≤ 25, 2-25-35, 3-≥ 35) Education (1-S1 Bachelors, 2-S2 Masters, 3-S3-PhD) Use of Internet (1-Personal use, 2-Studies, 3-Socializing, 4-All the above)

Table 1 displays demographic statistics for the three hundred and one (301) respondents. The sample was 27.9% male and 72.1% female. Most respondents were from Indonesia (71.1%), while28.9% were foreign students. Participants indicated their religion as Muslim (52.2%), Hindu (4.3%), Christian (36.5%) and Buddhist (7.0%). With respect to age, 56.1% were below 25, 39.9 % of respondents were between the ages of 25to 35, and only 4.0% of respondents were above 35 years of age. In regard to education level, 57.5 % of students were studying fora bachelor (S1) degree, 37.9% for masters (S2), and 4.7% for Ph.D. (S3). Use of internet was categorized as12% for personal use, 11.6% for studies, 26.6% for social media and social networking activities, while 49.8 % reported using the internet for all of the provided options.

Table 2 provides information on the validity of the variables and factor loadings (factor correlation coefficients). The coping strategies variable includes four factors (avoidance, problem-solving, religious coping, seeking solutions). Each factor loads on the coping strategies variable greater than .70, and an alpha coefficient greater than .90 suggests internal consistency. Positive psychology (PSY) and social capital (SC) are each measured with three items, all of which load between .59 to .79, and alpha coefficients of .857 and .955 (respectively) suggest high internal consistency. The technostress variable includes three factors (tech-complexity, tech-overload, tech-uncertainty). Each factor has a loading between .664 and .801, and an alpha coefficient greater than .90 suggests internal consistency. Overall, KMO and Bartlett's Test value also suggest the suitability of structure detection.

**Table 2:** Factor loading and Validity

Variables	Code	Factor Loading				ἀ		γ_s_		CR	(AVE)
Coping Strategies	AVD1	0.808				0.906	0.909		0.924		0.604
	AVD2	0.743									
	PS1	0.786									
	PS2	0.768									
	RC1	0.791									
	RC2	0.782									
	SS1	0.796									
	SS2	0.742									
Psychological and Social capital	PSY1			0.642		0.857	0.955		0.878		0.549
	PSY2			0.735							
	PSY3			0.592							
	SC1			0.799							
	SC2			0.760							
	SC3			0.881							
Techno Stress	TCX1				0.737	0.904	0.908		0.922		0.568
	TCX2				0.785						
	TCX3				0.751						
	TOL1				0.787						
	TOL2				0.801						
	TOL3				0.799						
	TUC1				0.767						
	TUC2				0.641						
	TUC3				0.701						
	Kaiser-Meyer-Olkin Measure of Sampling Adequacy							.918			
	Bartlett's Test of Sphericity			Approx. Chi-Square				4351.616			
				df				253			
				Sig.				.0000			

Note: AVD (avoidance), PS (Problem-solving), SS (seeking-support), RC (religious coping), PSY (positive psychology), SC (social capital), TCX (techno complexity), TOL (techno overload) TUC (techno uncertainty)

**Figure 1:**
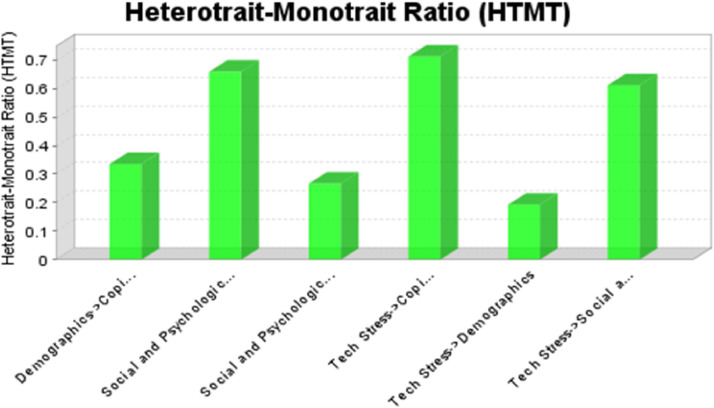
HTMT Graph

Evidence for discriminant validity is provided in Table 3; since all values are less than .85, this suggests discriminant validity exists between these constructs. In addition, Table 4 and Figure 1 show the results of HTMT analyses, which also help establish discriminant validity.

**Table 3:** Discriminant validity

		1	2	3	4
1	Coping Strategies	0.7773			
2	Demographics	-0.2823	0.4446		
3	PSY wellbeing and social capital	0.5982	-0.1763	0.7411	
4	Tech Stress	0.652	-0.1136	0.5829	0.7538

Note: Latent variable “demographics” comprised six variables i.e. Gender, Nationality, Religion, Age, Education and Use of internet as detailed in table 1

**Table 4:** HTMT

	1	2	3	4
1	Coping Strategies			
2	Demographics	0.3356		
3	PSY wellbeing and social capital	0.6587	0.267	
4	Tech Stress	0.7123	0.1935	0.6112

Note: Latent variable “demographics” comprised six variables i.e. Gender, Nationality, Religion, Age, Education and Use of internet as detailed in table 1

## 2 Experimental design, materials, and methods

The data were collected during the Spring 2018 semester from university students in Java province using a distributed online questionnaires survey research approach [2]. Respondents were required to answer all survey items; hence no missing data was reported. Consent was obtained from each participant. Demographic data was gathered from the respondents, as well as perceived technostress, coping strategies, psychological well-being, and social capital. The survey instrument appears in Supplementary Material.

Participants responded to items on a Likert scale, ranging from 1 (strongly disagree) to 5 (strongly agree). The questionnaires were taken from the extant literature [3], [4], [5] and can be found in the supplementary material. SPSS (v.25.0) and Smart-PLS (3.0) were used to generate descriptive statistics, correlations in Table 6, regression in Table 5, reliability, discriminant validity, and HTMT ratio.

**Table 5:** Regression model summary

Coefficients^a^	Std. Error	Beta	t	Sig.	Confidence Interval	
					Lower	Upper
(Constant)	1.335		4.618	***	3.538	8.793
TS ←Avoidance Strategy	0.262	0.038	0.582	0.561	-0.363	0.668
TS ← Seeking Support	0.260	0.045	0.694	0.488	-0.331	0.692
TS ← Problem Solving	0.289	0.340	4.719	***	0.794	1.931
TS ← Religious Coping	0.243	0.201	3.034	***	0.259	1.215
TS ← Positive Psychology	0.156	-0.059	-1.074	0.283	-0.475	0.140
TS ← Social Capital	0.136	0.264	5.043	***	0.418	0.952
R	0.700^a^					
R^2^	0.490					
F-Value (ANOVA)	47.02 (0.000)					
Sig ≤ 0.05						
Confidence Interval 95%						

a Dependent Variable: TS Note: TS (technostress)

**Table 6:** Correlation coefficients

		1	2	3	4	5	6	7	8	9
1	T Overload	1								
2	T Complexity	.737⁎⁎	1							
3	T Uncertainty	.718⁎⁎	.795⁎⁎	1						
4	Avoidance	.478⁎⁎	.486⁎⁎	.482⁎⁎	1					
5	Seeking Support	.463⁎⁎	.483⁎⁎	.488⁎⁎	.664⁎⁎	1				
6	Problem Solving	.586⁎⁎	.603⁎⁎	.554⁎⁎	.721⁎⁎	.719⁎⁎	1			
7	Religious Coping	.491⁎⁎	.561⁎⁎	.495⁎⁎	.623⁎⁎	.636⁎⁎	.673⁎⁎	1		
8	Psychological Wb	.317⁎⁎	.342⁎⁎	.319⁎⁎	.352⁎⁎	.388⁎⁎	.393⁎⁎	.565⁎⁎	1	
9	Social Capital	.436⁎⁎	.493⁎⁎	.492⁎⁎	.420⁎⁎	.394⁎⁎	.478⁎⁎	.443⁎⁎	.524⁎⁎	1

⁎⁎ Correlation is significant at the 0.01 level (2-tailed).

The measure of technostress [TS; [1], [3], [4]] used in this data includes three sub-constructs: technology overload, technology complexity, and technology uncertainty. Technology overload (TOL) was measured with three items and explains the increased nature of technology and its role in live of individuals (e.g., “I feel no escape from technology”). Technology complexity (TCX) was measured with three items and describes the emerging complexities due to the increased inclusion of technology (e.g., “working all day online is straining for me”). Technological uncertainty (TUC) was measured with three items and describes the rapid change of technology causes uncertainty (e.g., “I experience new technology development so often”).

The measure of coping strategies [5] used in this data includes four sub-constructs: avoidance, seeking support, problem-solving, and religious coping. Avoidance (AVD) was measured with two items, and measures the evasion of planning behavior (e.g., “I avoid doing things when I am stressed”). Seeking support (SS) was measured with two items and describes a personal plan of seeking some support in stress (e.g., “I talk about the situation because talking about it helped me feeling better”). Problem solving (PS)was measured with two items, and measures coping with stress through solving the problem (e.g., “I tried different ways to solve the problems until one that worked”). Religious coping (RS) was measured with two items, and explains the inclination to cope with stress through religion (e.g., “I saw my situation as God's will”)

Psychological well-being was measured with three items, and measures hopefulness and feeling good about oneself (e.g., “I take a positive attitude towards myself”). Social capital was measured with three items and explains cultural awareness and social cohesion with society (e.g., “I like attending cultural events with my friends”).

## Appendix Supplementary materials

Image, application 1

Image, application 2
