# Supplementary material for: Data set on coping strategies in the digital age: The role of psychological well-being and social capital among university students in Java Timor, Surabaya, Indonesia
Source: Data Brief. 2020 Apr 23;30:105583. doi: 10.1016/j.dib.2020.105583 (PMC7184248; doi:10.1016/j.dib.2020.105583)
Supplement: Supplementary file 2 [file mmc2.docx]

**Transparency document**

Supplementary data associated with this article can be accessed online: http://dx.doi.org/10.17632/jz42th6t4t.5
